# Survival from cancer of the breast in women in England and Wales up to 2001

**DOI:** 10.1038/sj.bjc.6604587

**Published:** 2008-09-23

**Authors:** M J Quinn, N Cooper, B Rachet, E Mitry, M P Coleman

**Affiliations:** 1 Social and Health Analysis and Reporting Division, Office for National Statistics (Room FG/114), 1 Myddelton Street, London EC1R 1UW, UK; 2 Cancer Research UK Cancer Survival Group, Non-Communicable Disease Epidemiology Unit, Department of Epidemiology and Population Health, London School of Hygiene and Tropical Medicine, Keppel Street, London WC1E 7HT, UK; 3 Département d'Hépatogastroentérologie et Oncologie Digestive, Centre Hospitalo-Universitaire Ambroise-Paré, 9 avenue Charles de Gaulle, F-92100 Boulogne, France

Breast cancer is the most common cancer in women worldwide (Parkin, 2001). In England and Wales at the end of the 1990s, approximately 36 000 new cases were diagnosed each year, representing 30% of the annual total of 120 000 cases. The number of cases exceeds the combined total for the second and third most common cancers in women, those of the large bowel (14 700 cases, 12%) and lung (12 600 cases, 10%). Breast cancer in men is comparatively uncommon, and is not considered further here.

During the 1970s and 1980s, age-standardised incidence increased on average by approximately 2% each year (Quinn *et al*, 2001). The NHS breast-screening programme began in 1988, covering women aged 50–64 years and using single mediolateral oblique view mammography and a 3-year interval between screens. During the prolonged prevalence round, overall breast cancer incidence increased by approximately 20%; it subsequently declined, but then rose again, and by the late 1990s, incidence was some 7% higher than at the peak in the early 1990s (Office for National Statistics, 2003). Part of the increase, possibly as much as 20% in post-menopausal women, may have resulted from the increasing use of hormone replacement therapy (HRT) in the 1990s (Beral *et al*, 1997).

After the exclusion of *in situ* tumours, we analysed the data for 382 277 women diagnosed with a first, primary, malignant neoplasm of the breast in England and Wales during the 14-year period 1986–1999 and followed up to 31 December 2001, some 89% of those eligible for analysis. Approximately 6.1% of women who were otherwise eligible were excluded with zero recorded survival (date of diagnosis same as date of death): most of these women will have been registered from a death certificate only (DCO), and their duration of survival is unknown, but they could not be reliably distinguished in these data from women with true zero survival. The proportion of women excluded from analysis as DCO records was very similar in all socioeconomic groups. A further 2.4% of women were excluded because it was not their first primary cancer: for one-third of these women, the previous cancer was also a breast cancer, diagnosed before 1986.

Major shifts in the morphological distribution of breast cancers have occurred over the last 30 years. The proportion described as an adenocarcinoma has been falling steadily, from 36% in the early 1970s (Coleman *et al*, 1999) to 6% in these data by the late 1990s. In the early 1970s, ductal, lobular and medullary tumours comprised less than 10% of all breast cancers, but by the late 1990s this figure had reached 75%, ductal carcinomas alone comprising 60% of all breast cancers. The proportion of tumours with unspecified morphology has continued to fall, from 47% in 1971–1975 to 30% in 1986–1990 and less than 5% in 1996–1999. Even this massive improvement in data quality cannot account for the overall increase in ductal, lobular and medullary tumours.

## Survival trends

**Figure 1:**
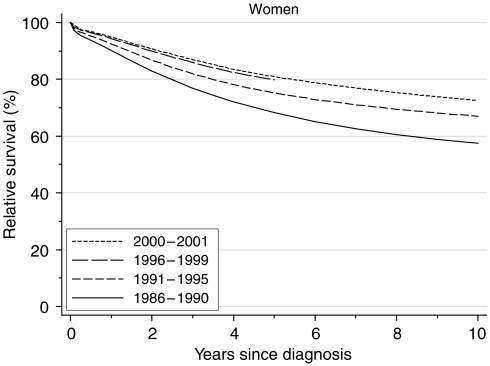
Relative survival (%) up to 10 years after diagnosis by calendar period of diagnosis: England and Wales, adults (15–99 years) diagnosed during 1986–1999 and followed up to 2001. Survival estimated with cohort or complete approach (1986–1990, 1991–1995, 1996–1999) or hybrid approach (2000–2001) (see Rachet *et al*, 2008).

For women diagnosed in the late 1990s, relative survival 1 year after diagnosis had reached 94%, a deprivation-adjusted average increase of 2.0% every 5 years since 1986–1990 (Table 1, Figure 1). Five-year survival had reached 80%, an average increase of 6.2% every 5 years over the same period. Ten-year survival rose even more markedly, by almost 10%, from 58% for women diagnosed during 1986–1990 to 67% for women diagnosed during 1991–1995, just 5 years later.

This trend is confirmed by hybrid analysis of the survival probabilities observed during 2000–2001 (Brenner and Rachet, 2004), with a predicted 10-year survival of 73% for women diagnosed at about that time.

## Deprivation

**Figure 2:**
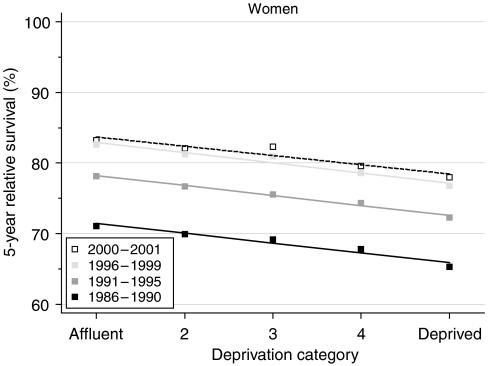
Trends in the deprivation gap in 5-year relative survival (%) by calendar period of diagnosis: England and Wales, adults (15–99 years) diagnosed during 1986–1999 and followed up to 2001.

The deprivation gap in 5-year and 10-year survival for women diagnosed during 1986–1990 was close to 6% (Table 2). The gap in 1-year survival was smaller, at approximately 3%. Survival has increased in all groups of the population, but the difference between rich and poor has not changed at all (Figure 2).

Short-term predictions of survival by socioeconomic group, again using hybrid analysis of the probabilities of survival observed during 2000–2001, do not suggest any imminent change in the significant deprivation gap in survival between affluent and deprived women.

## Comment

Breast cancer survival rose rapidly and significantly during the 1990s, and although the difference in survival between affluent and deprived women did not improve, at least it did not widen, in contrast to the widening deprivation gap in survival seen for most other malignancies (Coleman *et al*, 2004).

The trends in breast cancer survival among women diagnosed up to 1999 extend the continuing increase seen since the 1970s and 1980s, when 1-year survival rose from 84 to 90% and 5-year survival from 54 to 68% (Coleman *et al*, 1999). Women aged 50–64 years were invited for mammographic screening from 1988, and 5-year survival for women in the age range 50–69 years rose by 7–10% between 1986 and 1993, much more than for either older women (less than 2%) or younger women (less than 4%) (Coleman *et al*, 2000a, 2000b). By the late 1990s, however, this difference in age-specific trends had vanished (Rachet *et al*, 2005). Earlier diagnosis following mammography will increase survival time even if death is not delayed by treatment (lead-time bias), but the age-specific survival trends suggest that lead-time bias is unlikely to be the sole explanation for the overall increase in survival.

**Figure 3:**
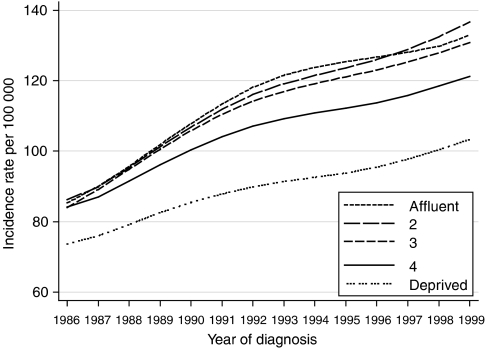
Trends in the age-standardised incidence of breast cancer in women aged 15–99 years, by deprivation group: England and Wales, 1986–1999.

Further analysis of our data shows that the incidence of breast cancer rose more rapidly among affluent women than among deprived women between 1986 and 1999 (Figure 3). If the more marked increase in incidence among affluent women were solely attributable to higher compliance with screening, and if we accept the impact of screening on survival, then the deprivation gap in relative survival would also have been expected to increase, and it did not (Figure 2). The incidence trends may therefore also reflect deprivation-specific trends in risk factors, including reproductive history and the use of HRT.

Despite the increase in breast cancer incidence during the 1990s, mortality has fallen in all age groups (Quinn *et al*, 2001). The larger falls in mortality in the screened age groups indicate that by the late 1990s, approximately one-third of the 20% overall reduction in breast cancer mortality was directly attributable to screening, although most of the fall in mortality was attributable to a combination of earlier diagnosis outside the screening programme, increasingly widespread use of tamoxifen since the 1980s and other, more recent improvements in chemotherapy, and more indirect effects of the screening programme, such as improvement in the organisation and delivery of services to symptomatically detected cases as well as screen-detected cases (Blanks *et al*, 2000).

We have recently predicted that long-term survival up to 20 years after diagnosis is likely to continue improving. The largest predicted increase also affects women aged 50–69 years at diagnosis (Woods *et al*, 2007).

For more than 30 years, breast cancer survival in England and Wales has been consistently lower in women from deprived areas, even after adjustment for higher background mortality in the more deprived areas. In the 1970s and early 1980s, the deprivation gap in 5-year survival was approximately 10%. The gap fell to 6% for women diagnosed during 1986–1990, but it has not changed any further since that time. This is remarkable because, for most cancers for which 5-year survival has improved as rapidly as it has done for breast cancer (approximately 6% every 5 years), the deprivation gap in survival has also widened considerably (Coleman *et al*, 2004). The deprivation gap in relative survival is adjusted for the wide socioeconomic differences in background mortality, and for changes over time in those differentials. Differences in crude survival (unadjusted for background mortality; data not shown) are actually wider than those for relative survival. The lack of improvement in the deprivation gap in relative survival is therefore not an artefact of failure to adjust for changes in background mortality among women with breast cancer.

## Figures and Tables

**Table 1:** Trends in relative survival (%) by time since diagnosis and calendar period of diagnosis: England and Wales, adults (15–99 years) diagnosed during 1986–1999 and followed up to 2001

		**Calendar period of diagnosis** a									
		**1986–1990**		**1991–1995**		**1996–1999**		**Average change (%) every 5 years** b		**Prediction**^c^ **for patients diagnosed during 2000–2001**	
**Time since diagnosis**		**Survival (%)**	**95% CI**	**Survival (%)**	**95% CI**	**Survival (%)**	**95% CI**	**Survival (%)**	**95% CI**	**Survival (%)**	**95% CI**
1 year	Women	**90.2**	(90.0, 90.4)	**92.5**	(92.3, 92.6)	**94.4**	(94.2, 94.5)	2.0**	(1.7, 2.3)	**94.9**	(94.7, 95.1)
5 years	Women	**68.3**	(68.0, 68.6)	**75.2**	(74.9, 75.4)	**79.8**	(79.5, 80.1)	6.1**	(5.5, 6.7)	**80.9**	(80.5, 81.3)
10 years	Women	**57.5**	(57.1, 57.8)	**67.0**	(66.7, 67.4)			9.6**	(8.4, 10.9)	**72.5**	(72.1, 73.0)

CI=confidence interval.

a Survival estimated with cohort or complete approach (see Rachet *et al*, 2008).

b Mean absolute change (%) in survival every 5 years, adjusted for deprivation (see Rachet *et al*, 2008).

c Survival estimated with hybrid approach (see Rachet *et al*, 2008).

^**^P<0.01.

**Table 2:** Trends in the deprivation gap in relative survival (%) by time since diagnosis and calendar period of diagnosis: England and Wales, adults (15–99 years) diagnosed during 1986–1999 and followed up to 2001

		**Calendar period of diagnosis^a^**									
		**1986–1990**		**1991–1995**		**1996–1999**		**Average change (%) every 5 years^b^**		**Prediction^c^ for patients diagnosed during 2000–2001**	
**Time since diagnosis**		**Deprivation gap (%)**	**95% CI**	**Deprivation gap (%)**	**95% CI**	**Deprivation gap (%)**	**95% CI**	**Deprivation gap (%)**	**95% CI**	**Deprivation gap (%)**	**95% CI**
1 year	Women	−3.1**	(−3.6, −2.5)	−2.9**	(−3.4, −2.5)	−2.6**	(−3.1, −2.2)	**0.2**	(−0.1, 0.6)	−2.7**	(−3.2, −2.1)
5 years	Women	−5.6**	(−6.5, −4.7)	−5.6**	(−6.4, −4.9)	−5.8**	(−6.7, −4.8)	**−0.1**	(−0.8, 0.6)	−5.2**	(−6.3, −4.1)
10 years	Women	−5.6**	(−6.6, −4.6)	−5.9**	(−6.9, −4.9)			**−0.3**	(−1.7, 1.2)	−6.1**	(−7.5, −4.8)

CI=confidence interval.

a Survival estimated with cohort or complete approach (see Rachet *et al*, 2008).

b Mean absolute change (%) in the deprivation gap in survival every 5 years, adjusted for the underlying trend in survival (see Rachet *et al*, 2008).

c Survival estimated with hybrid approach (see Rachet *et al*, 2008).

^**^P<0.01.
